# Expression of circulating miR-486 and miR-150 in patients with acute myocardial infarction

**DOI:** 10.1186/s12872-015-0042-0

**Published:** 2015-06-16

**Authors:** Rui Zhang, Chao Lan, Hui Pei, Guoyu Duan, Li Huang, Li Li

**Affiliations:** Department of emergency, The First Affiliated Hospital of Zhengzhou University, No.1 Jianshe Road, Zhengzhou, Henan 450052 China

**Keywords:** Acute myocardial infarction, MicroRNAs, Biomarker

## Abstract

**Background:**

With its high morbidity and mortality, acute myocardial infarction (AMI) places a major burden on society and on individual patients. Correct, early correct diagnosis is crucial to the management of AMI.

**Methods:**

In this study, the expression of circulating miR-486 and miR-150 was investigated in AMI patients and the two miRNAs were evaluated as potential biomarkers for AMI. Plasma samples from 110 patients with AMI (65 patients with ST-segment elevation myocardial infarction (STEMI) and 45 patients with non-ST-segment elevation myocardial infarction (NSTEMI)) and 110 healthy adults were collected. Circulating levels of miR-486 and miR-150 were detected using quantitative real-time PCR in plasma samples.

**Results:**

Results showed that the levels of miR-486 and miR-150 were significantly higher in AMI patients than in healthy controls. Receiver operating characteristic (ROC) curve analyses indicated that the two plasma miRNAs were of significant diagnostic value for AMI, especially NSTEMI. The combined ROC analysis revealed an AUC value of 0.771 in discriminating AMI patients from healthy controls and an AUC value of 0.845 in discriminating NSTEMI patients from healthy controls.

**Conclusion:**

Results indicated that the levels of circulating miR-486 and miR-150 are associated with AMI. They may be novel and powerful biomarkers for AMI, especially for NSTEMI.

## Background

Acute myocardial infarction (AMI) is the acute necrosis of myocardial tissue due to persistent and severe ischemia [1]. AMI is one of the most frequently occurring cardiovascular diseases and one of the leading causes of morbidity and mortality in both developed and developing countries [2–4]. With China’s aging population and the projected increase in the rate of this disease, it is estimated that 16 million people will suffer from AMI in 2020 and 23 million in 2030 [5]. Because it is the world’s largest developing country, China is challenged to provide care for its large and growing population of AMI patients [6]. AMI is separated into two categories based on changes seen in the electrocardiography (ECG): ST-segment elevation myocardial infarction (STEMI) and non-ST-segment elevation myocardial infarction (NSTEMI). In STEMI, the infarct-related artery is usually totally occluded by fibrin-rich clots, and immediate reperfusion therapy is the initial approach. In contrast, the initial conservative strategy or the initial invasive strategy can be taken in patients with NSTEMI whose infarct-related artery is partially occluded by platelet-rich clots. Prompt diagnosis is critical to controlling the development of AMI and initiating appropriate therapy to reduce the mortality rate and improve prognosis. ECG is significant to differentiate the two AMI types. However, ECG has several limitations. For example, normal findings do not exclude the possibility of AMI. NSTEMI patients are often misdiagnosed because they frequently lack typical symptoms and obvious elevated ST-segment in their ECG. At present, some conventional biomarkers, such as blood troponins, cardiac myoglobin and creatine kinase-MB (CK-MB) are wildly used for clinical diagnosis [7]. However, the search for novel biomarkers for AMI is ongoing.

In recent years, with advances in molecular biology and technology, nucleotide-based biomarkers that may enhance diagnostic or prognostic effectiveness have attracted considerable attention. MicroRNAs (miRNAs) are small, non-coding, cellular RNAs 17–27 nucleotides in length. By pairing with the 3’ UTR of target mRNAs, they act as sequence-specific regulators of gene expression through translational repression and transcript cleavage [8, 9]. Many studies suggest that miRNAs play crucial roles in a variety of essential biological processes, including proliferation, development, differentiation, and apoptosis [10]. Aberrant expression of miRNAs in tissues contributes to various diseases, such as cancer and cardiovascular disease [11–13]. In addition, many miRNAs are remarkably stable and readily detectable in the peripheral blood or plasma [14, 15]. The levels of circulating miRNAs are different in specific ways under specific pathological conditions [16–18]. This indicates that circulating miRNAs may be excellent candidate diagnostic and prognostic biomarkers of various diseases [19, 20]. Recently, it has been reported that the levels of several miRNAs such as miR-1, miR-133a, miR-208b, miR-499, and miR-328 in the blood and plasma alter during AMI, suggesting the diagnostic value of circulating miRNAs in early AMI [21–26].

It has been reported that miR-486 is a potent modulator in cardiac/skeletal muscle and miR-150 is involved in many cardiovascular diseases [27]. We also performed a preliminary plasma miRNA microassay chip analysis of AMI patients and healthy controls and the results showed significant changes in the levels of miR-486 and miR-150 in AMI patients which was in accordance with a recent study about serum miR-486 and miR-150 [28]. The purpose of the present study was to measure the levels of circulating miR-486 and miR-150 in AMI patients, so as to determine whether miR-486 and miR-150 could serve as novel biomarkers for the early diagnosis of AMI.

## Methods

### Patient characteristics

This study was approved by the Research Ethics Committee of Zhengzhou University, China, and the samples were collected with each patient’s informed consent.

One hundred and ten consecutive AMI patients were enrolled in this study from the First Affiliated Hospital of Zhengzhou University between June 2013 and May 2014. The clinical characteristics of all patients are given in Table 1. AMI patients were diagnosed using the following criteria: 1) acute ischemic chest pain; 2) abnormal electrocardiogram (pathological Q wave, ST-segment elevation, or depression); 3) levels of myocardial necrosis markers (troponins (cTns) and creatine kinase (CK)) more than twice the upper limit of the normal range. These patients were admitted to hospital no more than 24 h after the emergence of symptoms. Patients with previous MI or percutaneous coronary intervention (PCI), any hematological disease, acute or chronic infection, significant hepatic dysfunction, kidney failure (glomerular filtration rate (GFR) < 15 mL/min/1.73 m^2^ or on dialysis), or known or treated malignancies were excluded. In addition, 110 healthy adult volunteers (normal electrocardiograms and no history of cardiovascular diseases) were enrolled in this study. Five milliliter venous blood samples of patients with AMI were collected in EDTA anticoagulant tubes at admission. Samples were centrifuged at 3000 × *g* for 10 min at 4 °C, then the supernatant was isolated and centrifuged at 12,000 × *g* for 10 min at 4 °C. Plasma was collected and stored at −80 °C until RNA extraction.

**Table 1:** Clinical features, risk factors, and laboratory data of the cohort

Characteristics	All cohort (*n* = 220)	AMI cases (*n* = 110)		Control cases (*n* = 110)	*P*
		STEMI (*n* = 65)	NSTEMI (*n* = 45)		
Age (years)	57.99 ± 11.63	57.54 ± 12.07	57.93 ± 11.98	58.28 ± 11.32	0.712
Men/women (n/n)	170/50	51/14	36/9	83/27	0.520
Risk factors					
Hypertension (Y/N)	103/117	34/31	17/28	52/58	0.893
Hyperlipidemia (Y/N)	26/119	9/56	3/42	14/96	0.676
Diabetes mellitus (Y/N)	50/170	13/52	10/35	27/83	0.520
Smoking (Y/N)	47/173	15/50	8/37	24/86	0.869
Physical examination					
SBP (mmHg)	124.64 ± 18.89	125.88 ± 21.51	121.76 ± 16.35	124.23 ± 20.26	0.754
DBP (mmHg)	75.95 ± 11.58	76.68 ± 13.70	73.31 ± 9.60	77.30 ± 10.68	0.132
Heart rate (beats/ min)	74.10 ± 12.98	74.20 ± 13.03	75.38 ± 11.54	73.53 ± 13.57	0.511
Lab examination					
TC (mmol/L)	3.9232 ± 0.99	3.91 ± 1.07	3.92 ± 1.09	3.90 ± 0.91	0.998
TG (mmol/L)	1.3727 ± 0.80	1.48 ± 1.17	1.39 ± 0.88	1.30 ± 0.42	0.197
HDL (mmol/L)	1.0470 ± 0.22	0.98 ± 0.23	1.05 ± 0.27	1.09 ± 0.18	0.080
LDL (mmol/L)	2.5300 ± 1.00	2.52 ± 0.97	2.43 ± 0.87	2.58 ± 1.08	0.504
WBC (×10^9^/L)	8.589 ± 3.62	9.51 ± 4.13	10.02 ± 4.01	7.46 ± 2.68	<0.001
BUN (mmol/L)	5.9365 ± 2.73	6.25 ± 3.05	6.16 ± 2.64	5.66 ± 2.55	0.135
CK-MB (U/L)	35.68 ± 59.13	58.80 ± 90.75	48.46 ± 58.17	16.79 ± 8.63	<0.001
Cardiac troponin T (ng/mL)	0.68 ± 1.40	1.44 ± 1.89	1.16 ± 1.57	0.05 ± 0.12	<0.001
EF%	58.14 ± 7.83	56.35 ± 7.89	55.80 ± 8.28	60.15 ± 7.13	<0.001

*Abbreviations*: *AMI* acute myocardial infarction, *STEMI* ST-segment-elevation myocardial infarction, *NSTEMI* non-ST-segment elevation myocardial infarction, *SBP* systolic blood pressure, *DBP* diastolic blood pressure, *TC* total cholesterol, *TG* total triglyceride, *HDL* high-density lipoprotein, *LDL* low-density lipoprotein, *WBC* white blood cell, *BUN* blood urea nitrogen, *CK-MB* creatine kinase-MB, *EF* ejection fractions

Data are expressed as mean ± standard deviation. *P*: comparison between AMI patients with healthy controls

### RNA extraction

Total RNA was extracted from plasma using an miRNeasy Serum/Plasm Kit (Qiagen), in accordance with the manufacturer’s instructions. The RNA was dissolved in 20 μl of diethylpyrocarbonate-treated (DEPC-treated) water. The concentration and quality of the RNA samples were determined using NanoDrop2000c spectrophotometer (NanoDrop, Thermo Fisher Scientific, U.S.).

### Quantitative reverse transcription-polymerase chain reaction (qRT-PCR)

The levels of expression of miR-486 or miR-150 were quantified using quantitative real-time PCR (qRT-PCR) using TaqMan human microRNA assay kits (Applied Biosystems) according to the manufacturer’s instructions. The 15 μL RT reaction mix contains 0.3 μL of 100 mM dNTPs, 3 μL of MultiScribe Reverse Transcriptase (50 U/μL), 1.5 μL of 10 × RT buffer, 0.19 μL of RNase inhibitor (20 U/μL), 6 μL of RT primer, 3 μL of RNA sample and 1.01 μL of Nuclease-free water. For RNA from plasma samples, the concentration was diluted to 180 ng/μL. The reagent mixes were incubated at 16 °C for 30 min, 42 °C for 30 min and 85 °C for 5 min, the RT products were stored at – 20 °C until used for qRT-PCR. The 20 μL PCR reaction mix includes 1.0 μL 20 × TaqMan MicroRNA Assays, 0.16 μL RT product, 10 μL TaqMan Universal Master Mix II, No AmpErase UNG (2×), 8.84 μL Nuclease-free water. The PCR cycles consisted of an initial denaturation at 95 °C for 10 min, followed by 40 cycles of 95 °C for 15 s and 60 °C for 60 s. U6 snRNA served as an internal normalized reference. Each specimen was measured in triplicate. The relative expression values of each miRNA were calculated using the 2^-△^Ct method.

### Biochemical assays

The concentrations of plasma cardiac troponin T (cTnT) were measured using a Roche high-sensitivity assay performed on a Cobas C8000 system (Roche Diagnostics, Germany) with a detection limit of 0.003 μg/L, a 99th-percentile cutoff of 0.014 μg/L, and a CV of ≤10 % at 0.013 μg/L. CK-MB was also measured on Cobas C8000 instrument with a Roche IFCC-recommended method.

### Statistical analysis

Statistical treatment was performed using SPSS 20.0 software. All data were subjected to a normality test (Kolmogorov-Smirnov). Continuous data are presented as mean ± SD or median with interquartile range. Categorical variables are presented as counts and percentages. Independent sample t-tests and Mann–Whitney U tests were used to compare two groups of continuous variables and the chi-square test was used for categorical variables. Receiver operating characteristic (ROC) curve analysis and comparison of the derived area under the curve (AUC) were performed to assess miRNAs as predictors for distinguishing AMI from healthy controls. Multiple logistic regression analysis was carried out for evaluating the combined diagnostic accuracy of circulating miRNAs. The statistical significance was set at *P* < 0.05.

## Results

### Clinical characteristics of patients

Among 110 patients with AMI, 65 were diagnosed with STEMI and 45 were diagnosed with NSTEMI. Both the AMI group and control group were predominantly male (87/110 in the AMI group and 83/110 in the control group). The basic clinical characteristics of the patients in this study are shown in Table 1. There were no obvious differences in disease or personal history including hypertension, hyperlipidemia, diabetes, or smoking between AMI patients and control cases. Significant differences were observed between the two groups in white blood cells (WBC), CK-MB, cTnT, and the left ventricular ejection fraction (EF).

### Circulating miR-486 or miR-150 levels were significantly higher in AMI patients

**Fig. 1:**
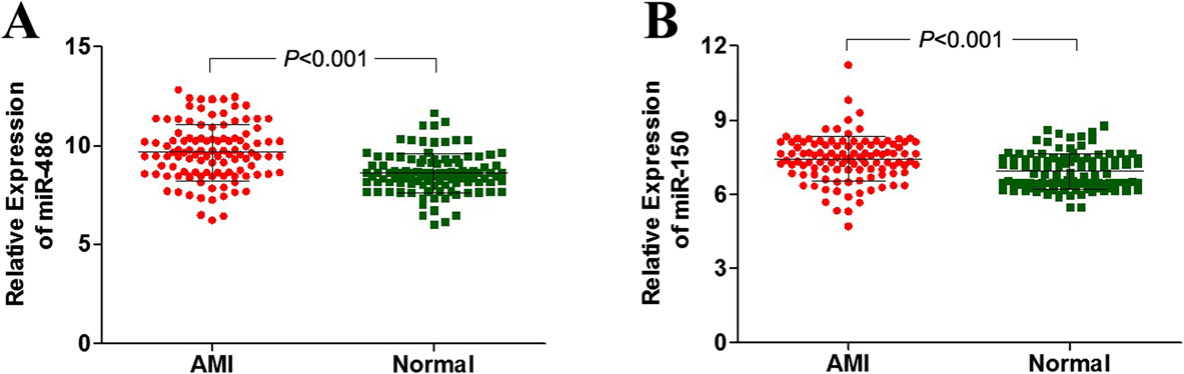
Expression of circulating miRNAs in AMI patients and control group. Plasma samples were collected upon admission no more than 24 h after AMI onset. **a**: The relative expression levels of miR-486 between AMI group and control group (*P* < 0.001). **b**: The relative expression levels of miR-150 between AMI group and control group (*P* < 0.001). Results were reported as mean ± SD

The blood samples of AMI patients were immediately collected after admission. We detected the miR-486 and miR-150 levels in plasma of 110 AMI patients (65 STEMI patients and 45 NSTEMI patients) and 110 non-AMI controls to assess the value of circulating miRNAs levels for predicting the onset of AMI. As shown in Fig. 1a, the plasma concentrations of miR-486 were markedly higher in AMI patients than in healthy controls (*P* < 0.001). In addition, the expression of miR-150 was markedly higher during the early phase, shortly after the occurrence of AMI in patients, than in controls (*P* < 0.001) (Fig. 1b).

### Circulating miR-486 and miR-150 expression levels as predictors of AMI

**Fig. 2:**
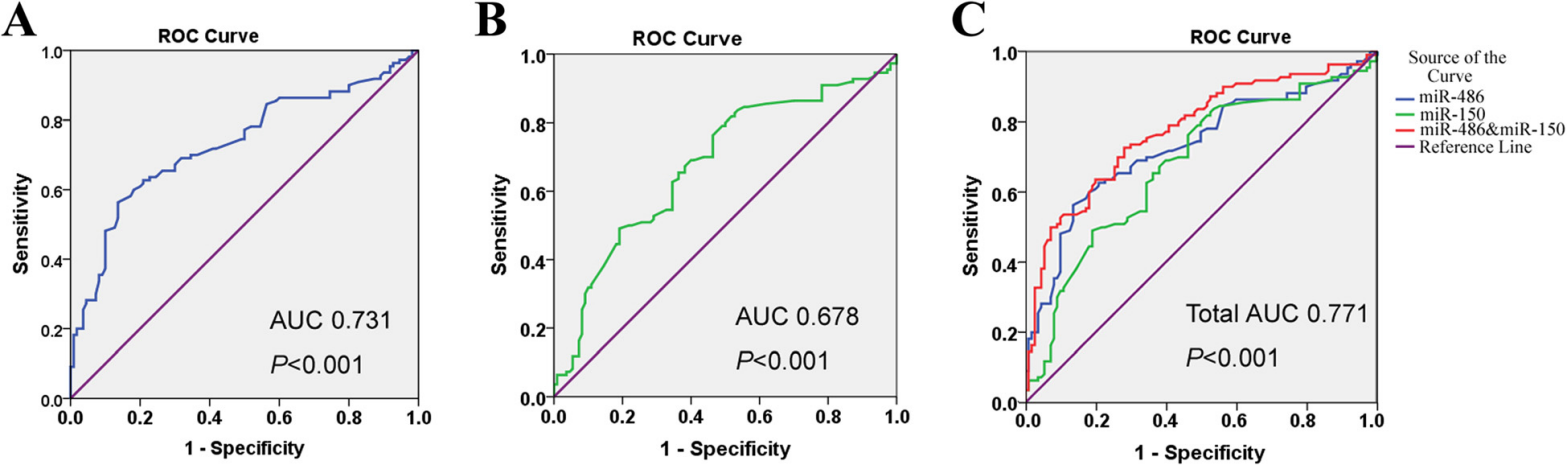
Receive operating characteristic (ROC) curves analyzed for the diagnostic value of circulating miRNAs. ROC curve for plasma (**a**) miR-486, (**b**) miR-150, and (**c**) the combination of the two miRNAs were able to distinguish AMI from the control group

To further evaluate the predictive power of circulating miR-486 and miR-150 for AMI, ROC curve and areas under ROC curve (AUC) analyses were performed. As shown in Fig. 2a and b, ROC curve analysis of miR-486 or miR-150 exhibited strong differentiation power between AMI patients and healthy controls during the early phase of AMI.

The AUC of miR-486 or miR-150 in AMI patients was 0.731 (*P* < 0.001), 0.678 (*P* < 0.001). Interestingly, the combination of the two miRNAs resulted in a higher AUC value of 0.771 (*P* < 0.001) than the AUC of miR-486 or miR-150 (Fig. 2a, b, c). These data suggested that the combination of circulating miR-486 and miR-150, which both had both high sensitivity and specificity, might be more suitable than miR-486 or miR-150 alone for diagnosing AMI.

### Expression pattern of miR-486 and miR-150 in STEMI and NSTEMI

**Fig. 3:**
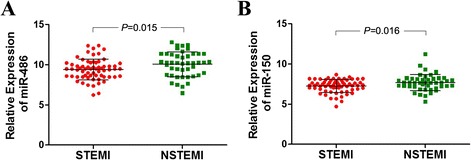
Relative expression of miRNAs in patients with STEMI and NSTEMI. The plasma levels of (**a**) miR-486 and (**b**) miR-150 were higher in the NSTEMI group than in the STEMI group. Results are reported as mean ± SD

**Fig. 4:**
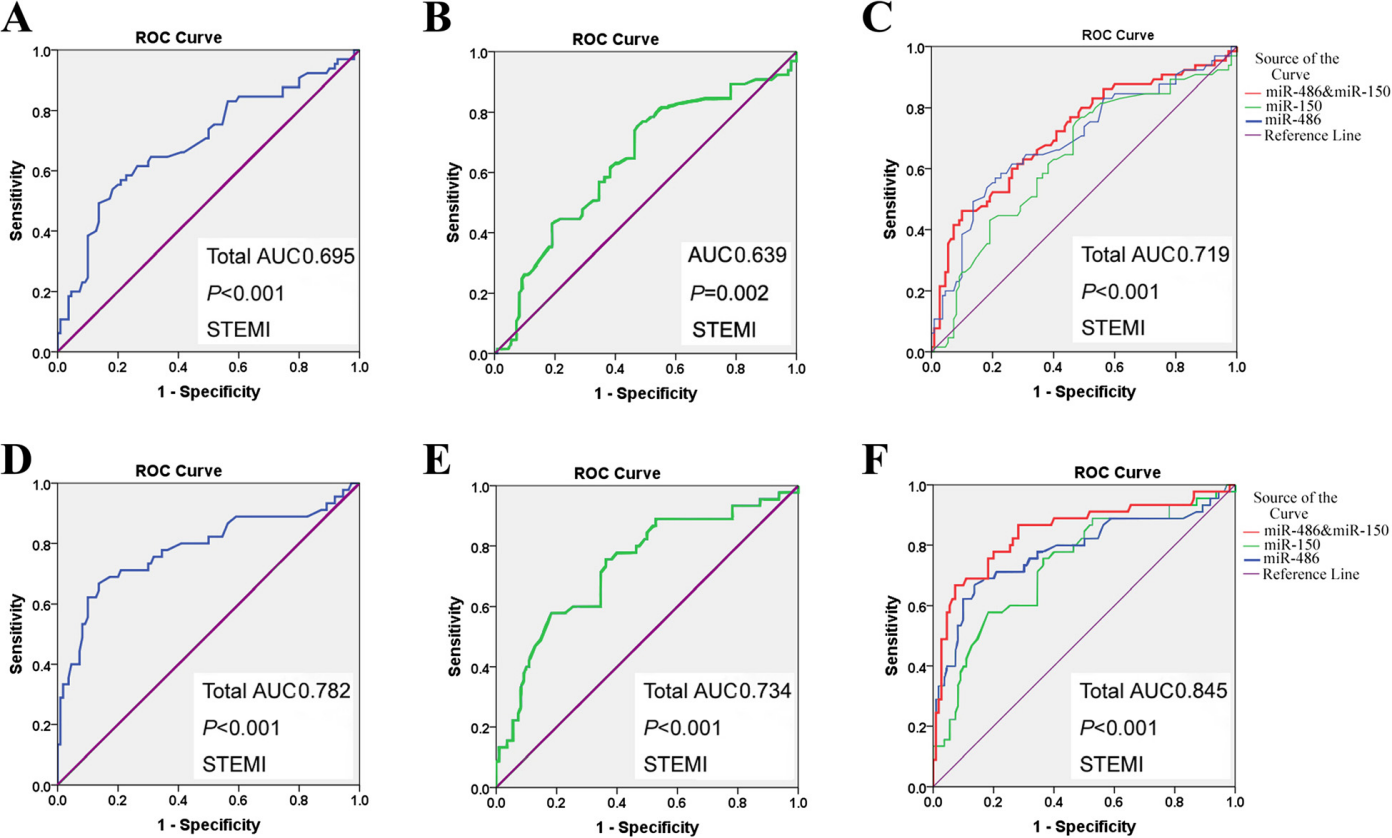
Evaluation of plasma microRNAs for the diagnosis of STEMI and NSTEMI by ROC curve analysis. The AUCs of plasma (**a**) miR-486, (**b**) miR-150, and (**c**) the combination of the two miRNAs were 0.695, 0.639, and 0.719 in the STEMI group. The AUCs of plasma (**d**) miR-486, (**e**) miR-150, and (**f**) the combination of the two miRNAs were higher (0.782, 0.734, and 0.845, respectively) in the NSTEMI group

A follow-up investigation was performed to determine whether the plasma miRNA levels in patients were associated with specific types of AMI. There was distinct difference between the level of plasma miR-486 (*P* = 0.015) and miR-150 (*P* = 0.016) between STEMI patients and NSTEMI patients (Fig. 3a and b). ROC curve analysis of miRNAs showed the AUC of plasma miR-486 and miR-150 in STEMI patients to be 0.695 and 0.639, respectively (Fig. 4a and b), which was lower than in NSTEMI patients (0.782, 0.734) (Fig. 4d and e). There was also a higher AUC value, 0.845, for the combination of miR-486 and miR-150 in the NSTEMI group than in the STEMI group (Fig. 4c and f). These data suggested miR-486 or miR-150 were highly sensitive and specific for the discrimination of NSTEMI cases from controls, and the combination of the two miRNAs was shown to predict NSTEMI with even higher power.

## Discussion

AMI remains an important cause of death in the world. Early and reliable diagnosis may prompt patients to undergo reperfusion therapy early and this may improve the survival rate of AMI patients.

Recent studies have revealed that miRNA are important regulators in the pathogenesis of many diseases. Results have demonstrated that miRNAs can be exported or released by cells and circulate in bloodstream; these are called circulating miRNAs [29]. Numerous circulating miRNAs are involved in AMI. These include cardiac-specific miRNAs (miR-208a) and non-cardiac-specific miRNAs (miR-126, miR-328, miR-134) [30–33]. On account of their tissue specificity, rapid release kinetics and stability in plasma, circulating miRNAs are considered promising biomarkers for detecting a large number of diseases, especially cardiovascular diseases. However, studies on the expression of circulating miRNAs in AMI patients are limited.

In this study, qRT-PCR results showed that plasma miR-486 and miR-150 were visibly overexpressed in 110 AMI patients. The ROC analyses showed that the two miRNAs might be suitable diagnostic markers of AMI. Here, the expression of miRNAs was measured in STEMI patients and NSTEMI patients. There was a distinct difference in levels of miR-486 and miR-150 expression between the STEMI and NSTEMI groups. The results of ROC analyses indicated that miR-486 and miR-150 are specific and sensitive for the early diagnosis of NSTEMI.

miR-486 is a muscle-enriched miRNA. It was found to be downregulated in several muscular diseases, such as Duchenne’s muscular dystrophy and denervation-induced muscle atrophy [34, 35]. Overexpression of miR-486 could lead to muscle hypertrophy. The upregulation of circulating miR-486 in AMI patients may be related to cardiac hypertrophy. miR-150, an miRNA related to inflammation, was reported to be implicated in the pathogenesis of various cardiovascular diseases [36–38]. A microarray screen of plasma samples showed reduced levels of miR-150 in patients with pulmonary arterial hypertension compared to healthy controls. Moreover, plasma miR-150 level in these patients was a significant predictor of survival [39]. miR-150 was also dysregulated in serum of patients with unstable angina pectoris. The diagnostic accuracy for unstable angina pectoris was obviously improved by applying the miRNA panel including miR-132, miR-150, and miR-186 [40]. Furthermore, miR-150 may inhibit cardiac structural and functional remodeling during ischemic injury partly by direct repression of the pro-apoptotic gene egr2 and p2x7r (pro-inflammatory ATP receptor) in cardiomyocytes [41]. In addition, the transcription factor c-Myb, NOTCH3 receptor, and nonmetastatic melanoma protein B were the potential targets of miR-150. Dysregulation of these two miRNAs may be associated with pathological conditions other than cardiac damage.

## Conclusion

In conclusion, results demonstrated that miR-486 and miR-150 levels to be significantly high in the plasma of AMI patients, both STEMI and NSTEMI, suggesting that circulating miR-486 and miR-150 might be responsible for the onset of AMI, especially NSTEMI. It is possible that miR-486 and miR-150 could be suitable biomarkers against AMI.

## Abbreviations

AMI: Acute myocardial infarction
STEMI: ST-segment elevation myocardial infarction
NSTEMI: Non-ST-segment elevation myocardial infarction

## References

[1] White HD, Chew DP (2008). Acute myocardial infarction. Lancet.

[2] Yusuf S, Reddy S, Ounpuu S, Anand S (2001). Global burden of cardiovascular diseases: part I: general considerations, the epidemiologic transition, risk factors, and impact of urbanization. Circulation.

[3] Sodha NR, Clements RT, Feng J, Liu Y, Bianchi C, Horvath EM (2009). Hydrogen sulfide therapy attenuates the inflammatory response in a porcine model of myocardial ischemia/reperfusion injury. J Thorac Cardiovasc Surg.

[4] Ueshima H, Sekikawa A, Miura K, Turin TC, Takashima N, Kita Y (2008). Cardiovascular disease and risk factors in Asia: a selected review. Circulation.

[5] Moran A, Gu D, Zhao D, Coxson P, Wang YC, Chen CS (2010). Future cardiovascular disease in china: markov model and risk factor scenario projections from the coronary heart disease policy model-china. Circ Cardiovasc Qual Outcomes.

[6] Yang G, Wang Y, Zeng Y, Gao GF, Liang X, Zhou M (2013). Rapid health transition in china, 1990–2010: findings from the global burden of disease study 2010. Lancet.

[7] Jaffe AS, Ravkilde J, Roberts R, Naslund U, Apple FS, Galvani M (2000). It's time for a change to a troponin standard. Circulation.

[8] Zamore PD, Haley B (2005). Ribo-genome: the big world of small RNAs. Science.

[9] Mattick JS, Makunin IV: Non-coding RNA. Hum Mol Genet 2006, 15 Sepc No 1:R17-29. 10.1093/hmg/ddl046 16651366

[10] Plasterk RH (2006). Micro RNAs in animal development. Cell.

[11] Small EM, Olson EN (2011). Prevasive roles of microRNAs in cardiovascular biology. Nature.

[12] Meltzer PS (2005). Cancer genomics: small RNAs with big impacts. Nature.

[13] Sayed D, Abdellatif M (2011). MicroRNAs in development and disease. Physiol Rev.

[14] van Rooij E, Liu N, Olson EN (2008). MicroRNAs flex their muscles. Trends Genet.

[15] Lagos-Quintana M, Rauhut R, Yalcin A, Meyer J, Lendeckel W, Tuschl T (2002). Identification of tissue-specific microRNAs from mouse. Curr Biol.

[16] van Empel VP, De Windt LJ, da Costa Martins PA (2012). Circulating miRNAs: reflecting or affecting cardiovascular disease?. Curr Hypertens Rep.

[17] Kloosterman WP, Plasterk RH (2006). The diverse functions of microRNAs in animal development and disease. Dev Cell.

[18] Mitchell PS, Parkin RK, Kroh EM, Fritz BR, Wyman SK, Pogosova-Agadjanyan EL (2008). Circulating microRNAs as stable blood-based markers for cancer detection. Proc Natl Acad Sci U S A.

[19] Gilad S, Meiri E, Yogev Y, Benjamin S, Lebanony D, Yerushalmi N (2008). Serum microRNAs are promising novel biomarkers. PLoS One.

[20] Chen X, Ba Y, Ma L, Cai X, Yin Y, Wang K (2008). Characterization of microRNAs in serum: a novel class of biomarkers for diagnosis of cancer and other diseases. Cell Res.

[21] Häntzsch M, Tolios A, Beutner F, Nagel D, Thiery J, Teupser D (2014). Comparison of whole blood RNA preservation tubes and novel generation RNA extraction kits for analysis of mRNA and MiRNA profiles. PLoS One.

[22] Zhou H, He XY, Zhuang SW, Wang J, Lai Y, Qi WG (2014). Clinical and procedural predictors of no-reflow in patients with acute myocardial infarction after primary percutaneous coronary intervention. World J Emerg Med.

[23] Long G, Wang F, Duan Q, Chen F, Yang S, Gong W (2012). Human circulating microRNA-1 and microRNA-126 as potential novel indicators for acute myocardial infarction. Int J Biol Sci.

[24] Bostjancic E, Zidar N, Stajer D, Glavac D (2010). MicroRNAs miR-1, miR-133a, miR-133b and miR-208 are dysregulated in human myocardial infarction. Cardiology.

[25] Xiao J, Shen B, Li J, Lv D, Zhao Y, Wang F (2014). Serum microRNA-499 and microRNA-208a as biomarkers of acute myocardial infarction. Int J Clin Exp Med.

[26] He F, Lv P, Zhao X, Wang X, Ma X, Meng W (2014). Predictive value of circulating miR-328 and miR-134 for acute myocardial infarction. Mol Cell Biochem.

[27] Chen D, Goswami CP, Burnett RM, Anjanappa M, Bhat-Nakshatri P, Muller W (2014). Cancer affects microRNA expression, release, and function in cardiac and skeletal muscle. Cancer Res.

[28] Hsu A, Chen SJ, Chang YS, Chen HC, Chu PH (2014). Systemic approach to identify serum microRNAs as potential biomarkers for acute myocardial infarction. Biomed Res Int.

[29] Zheng HW, Wang YL, Lin JX, Li N, Zhao XQ, Liu GF (2012). Circulating MicroRNAs as potential risk biomarkers for hematoma enlargement after intracerebral hemorrhage. CNS Neurosci Ther.

[30] Olivieri F, Antonicelli R, Capogrossi MC, Procopio AD (2013). Circulating microRNAs (miRs) for diagnosing acute myocardial infarction: an exciting challenge. Int J Cardiol.

[31] Recchioni R, Marcheselli F, Olivieri F, Ricci S, Procopio AD, Antonicelli R (2013). Conventional and novel diagnostic biomarkers of acute myocardial infarction: a promising role for circulating microRNAs. Biomarkers.

[32] Sayed AS, Xia K, Yang TL, Peng J (2013). Circulating microRNAs: a potential role in diagnosis and prognosis of acute myocardial infarction. Dis Markers.

[33] Kuwabara Y, Ono K, Horie T, Nishi H, Nagao K, Kinoshita M (2011). Increased microRNA-1 and microRNA-133a levels in serum of patients with cardiovascular disease indicate myocardial damage. Circ Cardiovasc Genet.

[34] Small EM, O'Rourke JR, Moresi V, Sutherland LB, McAnally J, Gerard RD (2010). Regulation of PI3-kinase/Akt signaling by muscle-enriched microRNA-486. Proc Natl Acad Sci U S A.

[35] Alexander MS, Casar JC, Motohashi N, Myers JA, Eisenberg I, Gonzalez RT (2011). Regulation of DMD pathology by an ankyrin-encoded miRNA. Skelet Muscle.

[36] Devaux Y, Vausort M, McCann GP, Zangrando J, Kelly D, Razvi N (2013). MicroRNA-150: a novel marker of left ventricular remodeling after acute myocardial infarction. Circ Cardiovasc Genet.

[37] Devaux Y, Vausort M, McCann GP, Kelly D, Collignon O, Ng LL (2013). A panel of 4 microRNAs facilitates the prediction of left ventricular contractility after acute myocardial infarction. PLoS One.

[38] Li X, Kong M, Jiang D, Qian J, Duan Q, Dong A (2013). MicroRNA-150 aggravates H2O2-induced cardiac myocyte injury by down-regulating c-myb gene. Acta Biochim Biophys Sin (Shanghai).

[39] Rhodes CJ, Wharton J, Boon RA, Roexe T, Tsang H, Wojciak-Stothard B (2013). Reduced microRNA-150 is associated with poor survival in pulmonary arterial hypertension. Am J Respir Crit Care Med.

[40] Zeller T, Keller T, Ojeda F, Reichlin T, Twerenbold R, Tzikas S (2014). Assessment of microRNAs in patients with unstable angina pectoris. Eur Heart J.

[41] Tang Y, Wang Y, Park KM, Hu Q, Teoh JP, Broskova Z, Ranganathan P, Jayakumar C, Li J, Su H, Tang Y, Ramesh G, Kim IM: MicroRNA-150 protects the mouse heart from ischemic injury by regulating cell death. *Cardiovasc Res* 2015 (in press). 10.1093/cvr/cvv121 PMC4447807 25824147

